# Enhanced hydrogen evolution reaction activity of hydrogen-annealed vertical MoS_2_ nanosheets†

**DOI:** 10.1039/c8ra01147h

**Published:** 2018-04-17

**Authors:** Mengci He, Fanpeng Kong, Geping Yin, Zhe Lv, Xiudong Sun, Hongyan Shi, Bo Gao

**Affiliations:** Institute of Modern Optics, Key Lab of Micro-optics and Photonic Technology of Heilongjiang Province, Key Laboratory of Micro-Nano Optoelectronic Information System, Ministry of Industry and Information Technology, Department of Physics, Harbin Institute of Technology Harbin 150001 China gaobo@hit.edu.cn; School of Chemistry and Chemical Engineering, Harbin Institute of Technology Harbin 150001 China; Department of Physics, Harbin Institute of Technology Harbin 150080 China; Collaborative Innovation Center of Extreme Optics, Shanxi University Taiyuan 03006 China shi.hong.yan@hit.edu.cn

## Abstract

Molybdenum disulfide (MoS_2_) is a promising electrocatalyst for hydrogen evolution reaction (HER), but only edges and S-vacancies are catalytic active sites for the HER. Therefore, it is crucial to increase edge sites and S-vacancies for enhancing the HER activity of MoS_2_. Here, we report an enhanced HER activity of MoS_2_ by combing vertical nanosheets and H_2_ annealing. Compared to horizontal MoS_2_ nanosheets, pristine vertical MoS_2_ nanosheets showed better HER activity due to a larger amount of edges. H_2_ annealing further enhanced the HER activity of vertical MoS_2_ nanosheets remarkably. Scanning electron microscopy (SEM), X-ray photoelectron spectra (XPS) and electrochemical impedance spectroscopy (EIS) were used to elucidate the enhanced HER activity by H_2_ annealing. SEM images showed that H_2_ annealing roughened the MoS_2_ edges, leading to more edge sites. XPS data revealed the smaller S : Mo ratio after H_2_ annealing, meaning more S-vacancies. Meanwhile, EIS measurements showed that charge transfer was accelerated by H_2_ annealing. These findings elaborated the H_2_ annealing induced enhancement of the HER activity, which were further confirmed by the subsequent re-sulfurization experiment.

## Abstract 2

Vertical configuration and H_2_ annealing enhanced the hydrogen evolution reaction activity of MoS_2_ nanosheets.

## Graphical abstract

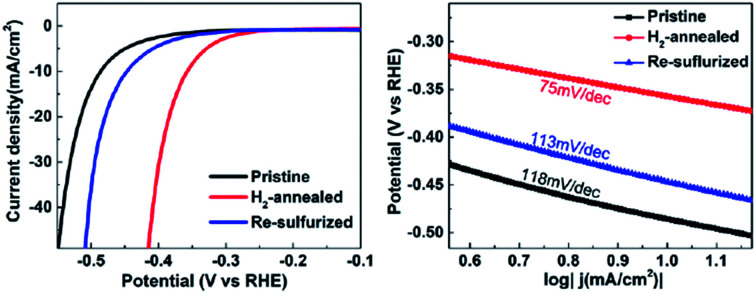

## Introduction

Because of its high efficiency, environment-friendliness and renewability, hydrogen is expected to play an important role in superseding the carbon-based fossil fuels. Sustainable, cost-effective and efficient production of hydrogen is a prerequisite for realizing hydrogen economy.^1,2^ Electrochemical catalytic hydrogen evolution reaction (HER) in acidic media is an efficient method to generate hydrogen from water splitting.^2^ Due to the presence of an overpotential, an electrolysis voltage is always needed, which leads to a waste of electric energy. Currently, there are no materials that could compare with platinum (Pt) in terms of the activity and stability. But its commercial application is greatly limited by the high cost and scarcity.^3,4^ Therefore, it highly demands to develop other efficient non-noble-metal HER electrocatalysts with high abundance and low cost to make H_2_ a competitive alternative energy source.^5,6^

A significant breakthrough was achieved when MoS_2_ was introduced as a promising and highly stable electrocatalyst for the HER.^7–19^ For decades, MoS_2_ was believed to be inactive of the HER, because the basal plane exhibits a hydrogen adsorption free energy of 1.92 eV.^20,21^ Recently, density functional theory (DFT) calculations on the Mo edge of MoS_2_ revealed that at 50% hydrogen coverage, it possessed a hydrogen adsorption free energy of 0.08 eV, near the optimal value of 0 eV.^7^ Soon after, it was experimentally confirmed that the edges of MoS_2_ are indeed the catalytic active sites for the HER.^9,10^ These studies motivated the development of MoS_2_ catalysts with a substantial fraction of further exposed edge sites, including hollow spheres,^9^ edge-exposed films,^22^ amorphous films,^23^ defect-rich films,^24^ nanodots^25^ and vertically aligned nanosheets.^8,24,26–28^ Therein, vertical MoS_2_ nanosheets, which have vertical channels for ion penetration and intimate contact between the active nanosheets and the random substrate, were shown to remarkably increase the density of edge sites and hence considered as an ideal geometry for improving the HER performance of MoS_2_-based HER catalysts.^9,22,29–41^

Although extensive efforts have been made to increase the number of edge sites, the overall HER activity is still limited, as generally only a small fraction of edge sites contribute to the reaction rate.^10,42^ Therefore, it is necessary and urgent to explore effective methods to increase the HER activity of inert basal plane sites in MoS_2_ nanosheets. Fortunately, S-vacancies in the basal plane of MoS_2_ nanosheets were recently explored and suggested to have a significant impact on the HER activity.^10,43–45^ By Ar or oxygen plasma exposure, or H_2_ treatment, S-vacancies were introduced into MoS_2_ nanosheets, and remarkably enhanced the HER activity.^44,46^ This method is only effective for flat MoS_2_ catalysts due to the Ar and oxygen plasma's directionality and thus unsuitable for vertical nanostructures and 3D nanostructures.^47^ Meanwhile, conductivity was also identified as a crucial factor for the HER activity, because a high conductivity ensures a fast charge transfer in the HER interface.^48–51^ Therefore, improving the conductivity while increasing active sites is the most promising but challenging task for optimizing the HER activity of MoS_2_ nanosheets.

Herein, we report an enhanced HER activity of MoS_2_ by combing vertical nanosheets and H_2_ annealing. Vertical MoS_2_ nanosheets were grown on glassy carbon by CVD method at 520 °C. Pristine vertical MoS_2_ nanosheets with a larger amount of edges showed better HER activity than horizontal ones. H_2_ annealing further enhanced the HER activity of vertical MoS_2_ nanosheets. Scanning electron microscopy (SEM), X-ray photoelectron spectra (XPS) and electrochemical impedance spectroscopy (EIS) were used to elucidate the enhanced HER activity by H_2_ annealing. SEM images showed that H_2_ annealing roughened the MoS_2_ edges, leading to more edge sites. XPS data revealed the smaller S : Mo ratio after H_2_ annealing, meaning more S-vacancies. Meanwhile, EIS measurements showed that charge transfer was accelerated by H_2_ annealing. These findings elaborated the H_2_ annealing induced enhancement of the HER activity, which were further confirmed by the subsequent re-sulfurization experiment.

## Experimental

### Growth of vertical MoS_2_ nanosheets by CVD method

**Fig. 1:**
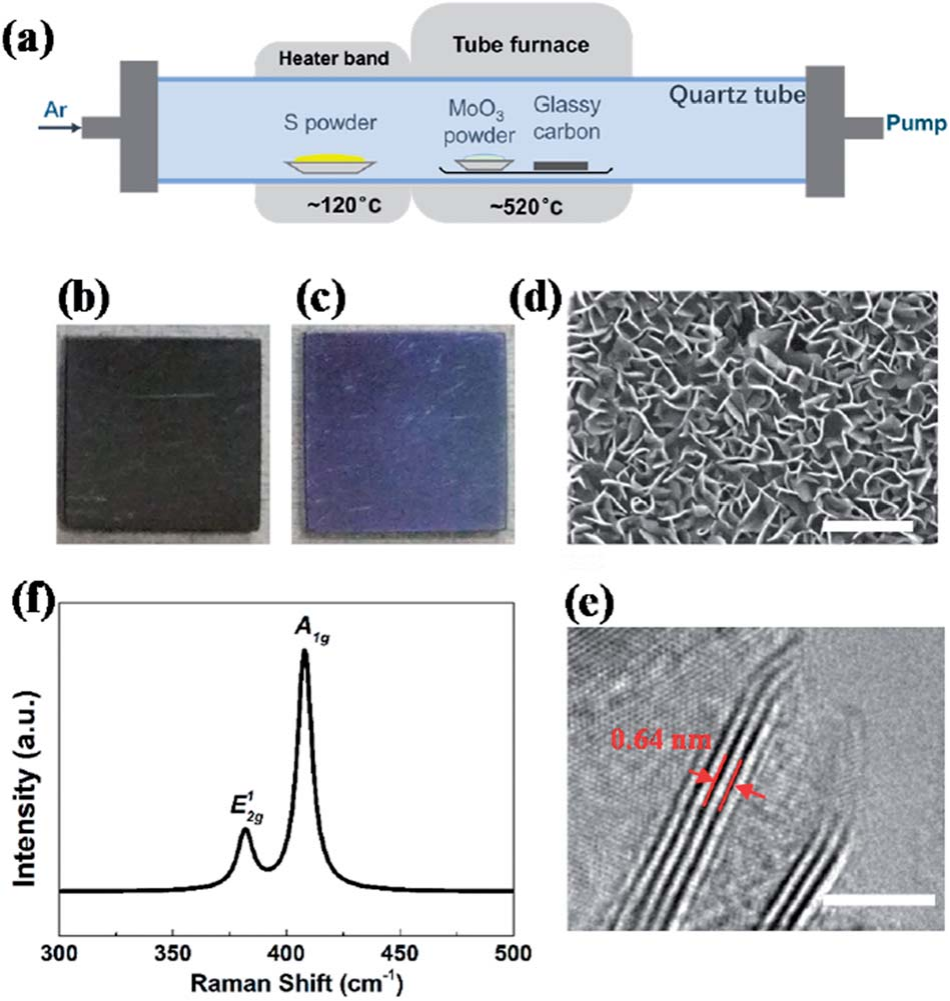
(a) Schematic diagram of dual-temperature zone CVD synthesis of vertical MoS_2_ nanosheets on glassy carbon. Photos of glassy carbon (b) without and (c) with vertical MoS_2_ nanosheets. (d) Typical SEM image of pristine vertical MoS_2_ nanosheets on glassy carbon; scale bar: 300 nm. (e) Typical HRTEM image of pristine vertical MoS_2_ nanosheets on glassy carbon with perfectly vertically aligned nanosheets consisting of a few layer with an interlayer spacing of 0.64 nm; scale bar: 5 nm. (f) Raman spectra of MoS_2_ nanosheets with two characteristic Raman vibration modes: in-plane vibration of molybdenum and sulfur atoms *E*_2g_^1^ and out-of-plane vibration of sulfur atoms *A*_1g_.

Vertical MoS_2_ nanosheets were synthesized by CVD method as shown in Fig. 1a. The vertical MoS_2_ nanosheets samples were grown on glassy carbon inside a tubular furnace equipped with 22 mm diameter quartz tube. A silica boat loaded with MoO_3_ powder (purity 99.95%) and glassy carbon substrate were placed one after another in the center of hot zone inside the tube furnace, and the distance between MoO_3_ powder and glassy carbon substrate was ∼2 cm. At the upstream of the tube, the other boat with sulfur powder (purity 99.5%) was placed outside the hot zone, which was mildly sublimated with heater band at ∼120 °C. Before heating, the tube was pumped down to a base pressure of ∼0.1 Pa and flushed with Ar to guarantee a favorable growth atmosphere. The furnace was then heated from room temperature to 520 °C at 50 °C min^−1^ and kept at 520 °C for 15 min with Ar flow of 10 sccm. After growth, the furnace was opened directly for rapid cooling to the room temperature with Ar flow of 200 sccm.

### Hydrogen annealing of vertical MoS_2_ nanosheets

Before the hydrogen annealing experiment, high quality vertical MoS_2_ nanosheets sample on glassy carbon was selected by electrochemical measurements. After putting selected MoS_2_ nanosheets sample into the quartz tube at center of the furnace, the temperature of the furnace was raised up to 780 °C at 50 °C min^−1^ with Ar flow of 50 sccm. After the temperature reached 780 °C, Ar was immediately switched to H_2_ with flow of 20 sccm, which is thought as the start point of the H_2_ annealing. After a desired time (5, 10, 15, 20, 25, 30 and 35 min, respectively), H_2_ was immediately switched to Ar with flow of 50 sccm and the furnace was shut off, leaving to cool down naturally. The annealing was carried out at low pressure of ∼55 Pa.

### Re-sulfurization of H_2_-annealed MoS_2_ nanosheets

The re-sulfurization was conducted following a similar process of growth experiment, except for the absence of MoO_3_ and longer treatment time. H_2_-annealed MoS_2_ sample was placed in the center of the furnace and excessive sulfur powder was put in the center of the heater bend with temperature of ∼120 °C. Before heating, the tube was pumped down to a base pressure of ∼0.1 Pa and flushed with Ar to guarantee a favorable growth atmosphere. The furnace was then heated from room temperature to 520 °C at 50 °C min^−1^ and kept at 520 °C for 30 min with Ar flow of 10 sccm. After re-sulfurization, the furnace was opened directly for rapid cooling to the room temperature with Ar flow of 200 sccm.

### Structural characterization

Surface morphology and structure were characterized by SEM and high resolution transmission electron microscopy (HRTEM, Tecnai TF20) techniques, respectively. SEM characterization was carried out on a Hitachi SU8000 instrument with an accelerating voltage of 15 kV and current of 10 μA. HRTEM samples were prepared by gently rubbing the TEM grid across the face of the MoS_2_ thin film to detach nanosheets and promote their adhesive on to the lacey-carbon TEM grid. Raman spectroscopy was measured using a B&W-Tek confocal Raman microscope with a laser excitation energy of 532 nm (2.33 eV). XPS were recorded on an ESCALAB MKII using an Al Kα excitation source.

### Electrochemical measurements

The experiment of HER was carried out by measuring the current correlated with the water dissociation in the ambient conditions. All of the electrochemical measurements were performed in an electrochemical workstation CHI604B with a standard three-electrode configuration. The working electrode was the vertical MoS_2_ nanosheets on glassy carbon. A graphite rod and an Ag/AgCl electrode were used as the counter electrode and the reference electrode, respectively. The performance of the HER was measured using linear sweep voltammetry from 0 to −0.6 V (*vs.* RHE) with scan rate of 5 mV s^−1^ at room temperature, which was conducted in 0.5 M H_2_SO_4_ aqueous solution. All the potentials were referenced to reversible hydrogen electrode (RHE) by the following equation: *E*(*V vs.* RHE) = *E*(*V vs.* Ag/AgCl) + 0.197 V + 0.059 pH. In 0.5 M H_2_SO_4_, *E*(*V vs.* RHE) = *E*(*V vs.* Ag/AgCl) + 0.197 V. An iR correction was normally employed to compensate for any potential loss arising from the external resistance of the electrochemical system. The electrolyte resistance and capacitance of the electrocatalysts were characterized by EIS. The ac impedance is measured at overpotential of −0.3 V (*vs.* RHE) within the frequency range from 0.1 to 10^6^ Hz with a perturbation voltage amplitude of 10 mV. Cyclic voltammetry (CV) was conducted from 0 to −0.6 V (*vs.* RHE) at 100 mV s^−1^ to investigate the cycling stability.

## Results and discussion

Fig. 1a shows an illustration of the dual-temperature zone CVD synthesis of vertical MoS_2_ nanosheets on glassy carbon. In the dual-temperature approach, heater band is isolated from the tube furnace. Therefore, the temperature of the heater band can be controlled independently from the temperature of the tube furnace. A 1 g portion of S powder was placed upstream of the gas flow in the quartz tube, right in the center of the heater band. 5 mg of MoO_3_ powder and glassy carbon was put in the center of the tube furnace. The S powder was heated to 120 °C in order to be evaporated by heater band. And the S vapor was carried downstream by Ar. At low pressure, S and MoO_3_ vapor reacted and formed MoS_2_ on glassy carbon at 520 °C, which is much lower than previous study.^22,43^

After the growth, the whole substrate turned violet from black (shown in Fig. 1b and c), indicating the formation of uniform MoS_2_ nanostructures. The morphology and structure of MoS_2_ were characterized by SEM and HRTEM, respectively. Fig. 1d shows the SEM image of vertical MoS_2_ nanosheets on glassy carbon. It can be seen that the size of MoS_2_ nanosheets is about 100–200 nm. From the HRTEM image in Fig. 1e, it can be seen that the nanosheets consisted of 4–5 layers with layer distance of 0.64 nm. Raman measurements were also performed to further confirm the presence of MoS_2_. As shown in Fig. 1f, there are two dominant Raman peaks at 382 and 408 cm^−1^, respectively, which originate from the in-plane *E*^1^_2g_ and out-of-planes *A*_1g_ vibrational modes of MoS_2_, respectively.

**Fig. 2:**
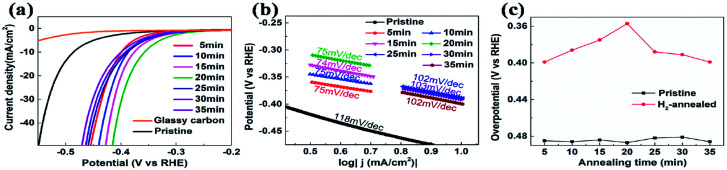
(a) Cathodic polarization curves at scan rate of 5 mV s^−1^ in 0.5 M H_2_SO_4_ and corresponding (b) Tafel plots. (c) Overpotential at 10 mA cm^−2^ of pristine (black squares) and H_2_-annealed (red dots) vertical MoS_2_ nanosheets *versus* annealing time.

The electrocatalytic HER activities were investigated in 0.5 M H_2_SO_4_ solution by linear sweep voltammetry (LSV) using a three-electrode setup at a scan rate of 5 mV s^−1^. An iR correction was normally employed to compensate for any potential loss arising from the external resistance of the electrochemical system. Fig. 2a shows the cathodic polarization curves of glassy carbon with and without vertical MoS_2_ nanosheets, which are indicated by black and orange lines, respectively. It can be seen that vertical MoS_2_ nanosheets exhibited great HER activity, compared to bare glassy carbon. A variety of methodologies have been proposed to evaluate the HER activity. Here overpotential at current density of 10 mA cm^−2^,^42^ onset overpotential and Tafel slope were used. The overpotential for vertical MoS_2_ nanosheets at current density of 10 mA cm^−2^ is 485 mV, which is lower than horizontal MoS_2_ nanosheets.^46,52^ The improvement is due to more edges in the vertical MoS_2_ nanosheets sample, and hence more active sites.^22^Fig. 2b shows the Tafel plot of pristine vertical MoS_2_ nanosheets, indicated by black dotted lines. The Tafel slope is determined by fitting the linear portion of the Tafel plot to the Tafel equation *η* = *b* log |*j*| + *a*, where *j* is the current density, *b* is the Tafel slope. The value of 118 mV dec^−1^ was obtained from the pristine sample. The pristine MoS_2_ sample exhibited the current density 1 mA cm^−2^ at the onset overpotential (301 mV), which was determined from Tafel plot as shown in Fig. S1.†

To further enhance HER activity, pristine vertical MoS_2_ nanosheets were annealed in H_2_ atmosphere, which has been demonstrated to be catalytically effective for horizontal MoS_2_ nanosheets.^46^ But the mechanism was not clear. Before H_2_ annealing, the cathodic polarization curves of each vertical MoS_2_ nanosheets sample were measured to assure that all pristine samples had similar HER activity (the overpotentials at current density of 10 mA cm^−2^ are shown by black squares in Fig. 2c). In the H_2_ annealing experiment, the selected MoS_2_ samples were placed in the center of the tube furnace set at 780 °C. For time-dependent H_2_ annealing, the different pristine samples were treated for 5, 10, 15, 20, 25, 30 and 35 min, respectively. To precisely control the H_2_ annealing time, 20 sccm of H_2_ only flowed when the temperature reached 780 °C, while 50 sccm of Ar flowed during the warming-up and cooling-down process.

Fig. 2a shows the cathodic polarization curves of pristine and H_2_-annealed vertical MoS_2_ nanosheets for 5, 10, 15, 20, 25, 30 and 35 min, respectively. It can be seen that all H_2_-annealed MoS_2_ samples had a smaller onset overpotential and a smaller overpotential at current density of 10 mA cm^−2^. Meanwhile, the onset potential and the overpotential at current density of 10 mA cm^−2^ have a relation to the annealing time. So, we plotted the overpotential at current density of 10 mA cm^−2^ as a function of annealing time (shown by red dots in Fig. 2c). It can be seen that, as the annealing time increased to 20 min, the overpotential decreased to 357 mV from ∼485 mV for pristine MoS_2_ samples, which is reproducible (see Fig. S3 of ESI†). The overpotential was increased when further increasing the annealing time, but it was still smaller than that of pristine MoS_2_ samples. The annealing time dependence of cathodic polarization curves also indicates good reproducibility of our method. Fig. 2b shows the Tafel plots of pristine and H_2_-annealed vertical MoS_2_ nanosheets for 5, 10, 15, 20, 25, 30 and 35 min, respectively. It can be seen that, when annealing for 5–20 min, all samples exhibited similar Tafel slopes of ∼75 mV dec^−1^, which are much lower than pristine samples with Tafel slopes of ∼118 mV dec^−1^. But when further increasing the annealing time, the Tafel slopes were increased to ∼102 mV dec^−1^. The HER results indicated that the HER activities of vertical MoS_2_ nanosheets were enhanced after H_2_ annealing, and was maximized with annealing time of 20 min, which showed better performance than CVD-grown vertical MoS_2_ nanosheets and H_2_ annealed horizontal MoS_2_ in previously reported.^22,46^

**Fig. 3:**
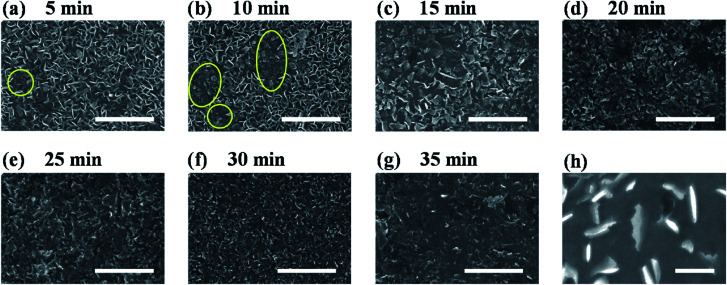
(a–g) SEM images of H_2_-annealed vertical MoS_2_ nanosheets for 5, 10, 15, 20, 25, 30 and 35 min, respectively; scale bar: 1 μm. (h) High magnification SEM image of H_2_-annealed vertical MoS_2_ nanosheets for 20 min, showing rough edges induced by H_2_ annealing; scale bar: 200 nm.

To figure out the origin of the enhanced HER activity of vertical MoS_2_ nanosheets by H_2_ annealing, the morphologies of H_2_-annealed samples were characterized by SEM. Fig. 3a–g show the SEM images of H_2_-annealed vertical MoS_2_ nanosheets samples for 5, 10, 15, 20, 25, 30 and 35 min at 780 °C, respectively. It can be seen in Fig. 3a that, when the annealing time is 5 min, only a few vertical MoS_2_ nanosheets were damaged (indicated by yellow circles), while most ones were intact. When further increasing the annealing time, the damaged MoS_2_ nanosheets were fragmentized into smaller nanosheets (indicated by yellow circles in Fig. 3b) and further formed aggregates (shown in Fig. 3c). After annealing for 20 min, almost all MoS_2_ nanosheets were damaged (shown in Fig. 3d). When the annealing time was increased to 35 min, all nanosheets were melted and the sheet size was greatly reduced (shown in Fig. 3g). It is particularly noted in Fig. 3h that the edge of the nanosheets became rough, indicating that H_2_ annealing increased the edges and hence active sites.

**Fig. 4:**
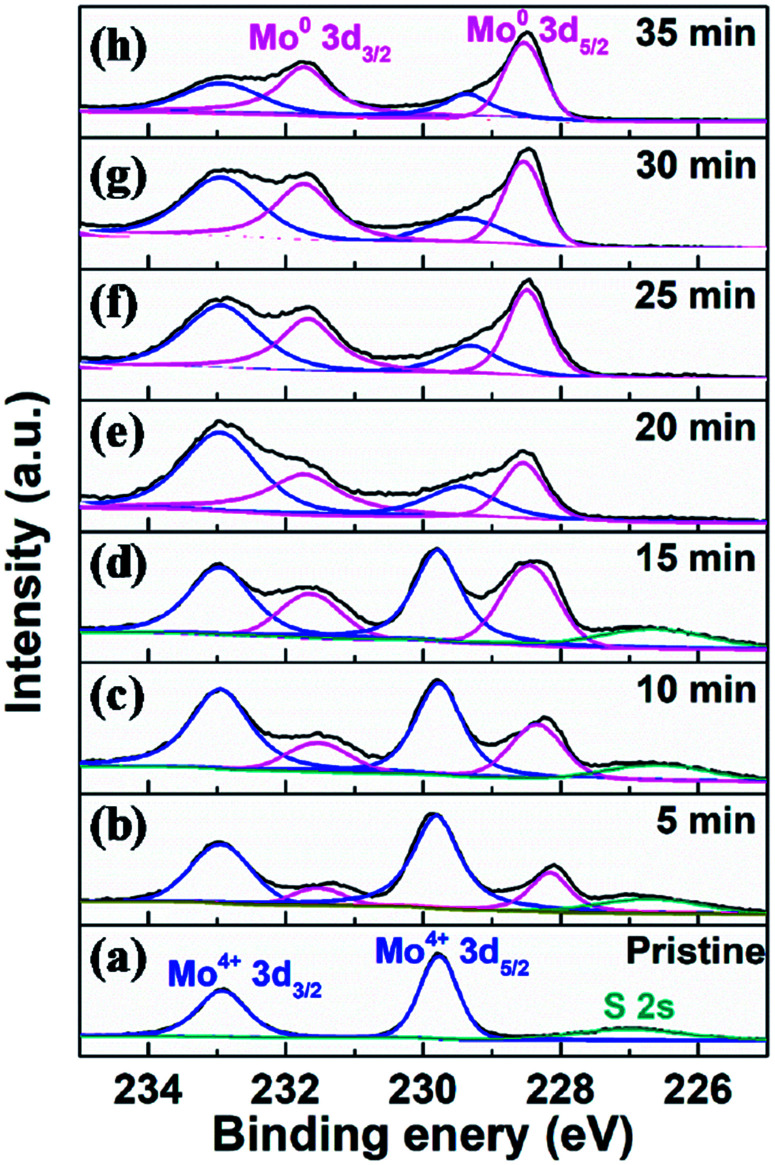
XPS data of Mo 3d region for (a) pristine and H_2_-annealed vertical MoS_2_ nanosheets for (b) 5, (c) 10, (d) 15, (e) 20, (f) 25, (g) 30 and (h) 35 min, respectively.

To fully elaborate the enhanced HER activity of vertical MoS_2_ nanosheets by H_2_ annealing, we also did the XPS characterization on the pristine and H_2_-annealed samples to explore the chemical states of the vertical MoS_2_ nanosheets. Fig. 4 shows the XPS data of Mo 3d region for the pristine and H_2_-annealed vertical MoS_2_ nanosheets for 5, 10, 15, 20, 25, 30 and 35 min, respectively. As shown in Fig. 4a, the spectrum of the pristine sample is dominated by a doublet with two sharp peaks, which are Mo 3d_5/2_ with binding energy of 229.7 eV and Mo 3d_3/2_ with binding energy of 232.9 eV. The distance between the two peaks was ∼3.2 eV, which corresponds to the formation of MoS_2_. The peak located at 226.5 eV corresponds to S 2s in MoS_2_.^53^

As shown in Fig. 4b, after H_2_ annealing for 5 min, a new doublet appeared in the Mo 3d region located at a lower binding energy to that of the original Mo^4+^ doublet of the pristine sample. The two components of the new doublet at 228.1 and 231.5 eV, could be assigned to Mo 3d_5/2_ and Mo 3d_3/2_ of Mo^0^, respectively, as in elementary molybdenum, which was reduced from the pristine MoS_2_ nanosheets by H_2_ annealing. The appearance of elementary molybdenum indicates the loss of sulfur and hence the appearance of S-vacancies in H_2_-annealed MoS_2_ samples. From the relative intensity between the new Mo^0^ doublet and the original Mo^4+^ doublet in Fig. 4b, the percentage of S-vacancies was determined to be 22.8%. As the annealing time was increased, the peak intensities of the new Mo^0^ doublet were increased, and so did the relative intensities between the new Mo^0^ doublet and the original Mo^4+^ doublet. It is also determined that the percentage of S-vacancies were 29.2%, 39.2%, 41.5%, 50.0%, 52.4% and 61.2% for H_2_-annealing time of 10, 15, 20, 25, 30 and 35 min, respectively. As shown in Fig. 2, the maximal HER activity was found at H_2_ annealing time of 20 min, corresponding to 41.5% of S-vacancies. Recent calculation showed that the optimal hydrogen adsorption free energy for HER occurred for an S-vacancy concentration that is between 12.5 and 15.62% of the surface atoms,^44,47^ which is much lower than our experiment result. It is known that besides hydrogen adsorption free energy, conductivity is also a crucial factor in HER, which we think is responsible for the high percentage of S-vacancies. Meanwhile, the intensity of the S 2s peak was gradually decreased without changing its shape and binding energy, which also suggested the production of S-vacancies in the H_2_-annealed MoS_2_ sample.

**Fig. 5:**
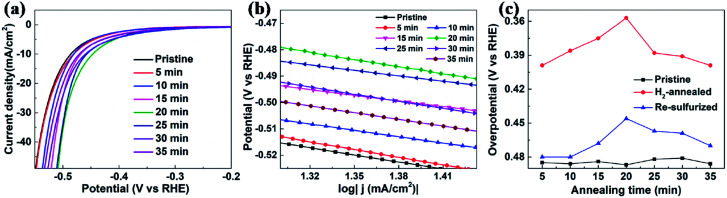
(a) Cathodic polarization curves at scan rate of 5 mV s^−1^ in 0.5 M H_2_SO_4_ and corresponding (b) Tafel plots. (c) Overpotential at 10 mA cm^−2^ of pristine (black squares), H_2_-annealed (red dots) and re-sulfurized (blue triangles) MoS_2_ nanosheets *versus* annealing time.

To further confirm that it is the S-vacancy that contributed to the enhanced HER activity of H_2_-annealed vertical MoS_2_ nanosheets, we re-sulfurized the H_2_-annealed MoS_2_ samples to repair the S-vacancies and measured the catalytic activities after the re-sulfurization. The re-sulfurization was achieved in the dual-temperature zone CVD with similar parameters to the synthesis of the vertical MoS_2_ nanosheets, except for the absence of MoO_3_ and longer treatment time. Fig. 5a shows the cathodic polarization curves of pristine and re-sulfurized MoS_2_ samples which were previously H_2_-annealed for 5, 10, 15, 20, 25, 30 and 35 min, respectively. It can be seen that, compared to H_2_-annealed MoS_2_ samples, both onset overpotential and overpotential at current density of 10 mA cm^−2^ were increased after re-sulfurization, but smaller than pristine MoS_2_ samples, which means HER activity were decreased after re-sulfurization, but still better than pristine MoS_2_ samples. Since the re-sulfurization filled all S-vacancies induced by H_2_ annealing (see XPS data of re-sulfurized vertical MoS_2_ nanosheets in Fig. S2 of ESI†), the decrease of the HER activity after re-sulfurization could be attributed to the reduction of S-vacancies. For the partial recovery of HER activities compared to pristine MoS_2_ samples, we conjecture that H_2_ annealing induced rough MoS_2_ edges played a role, which also could explain the dependence of the overpotential at current density of 10 mA cm^−2^ as on annealing time for re-sulfurized MoS_2_ samples (indicated by blue triangles in Fig. 5c).

**Fig. 6:**
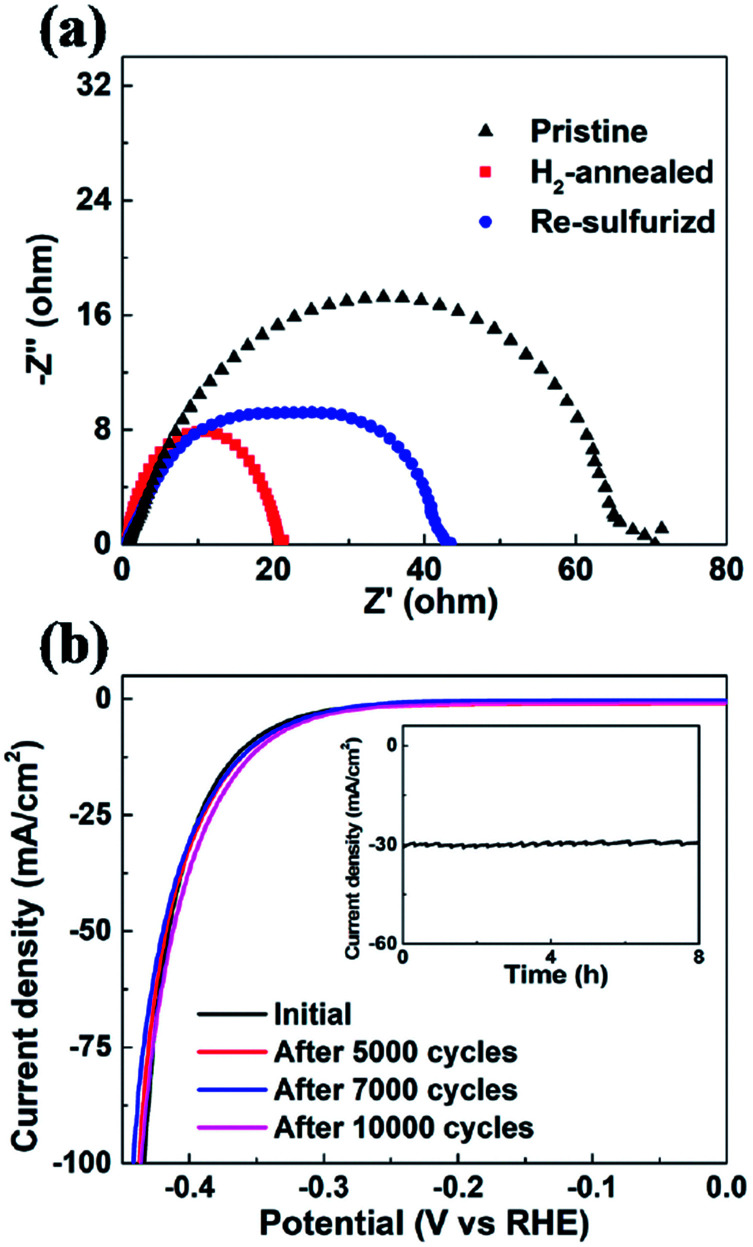
(a) EIS of pristine, H_2_-annealed and re-sulfurized vertical MoS_2_ nanosheets. All of the spectra were collected by scanning from 0.1 to 10^6^ Hz with an overpotential of 0.3 V. (b) Cathodic polarization curves of the H_2_-annealed MoS_2_ for 20 min before and after 5000, 7000 and 10 000 cycles, respectively. Inset: time-dependent current density of the H_2_-annealed MoS_2_ for 20 min under a static overpotential of 400 mV for 8 h.

In order to evaluate the kinetics of charge transfer in these samples, EIS measurements were carried out in a traditional three-electrode system. The diameter of the semicircle is considered to be related to charge transfer at the interface of the HER. A smaller diameter corresponds to more efficient charge transfer and smaller conductivity. Fig. 6a shows the Nyquist plots of pristine, H_2_-annealed and re-sulfurized MoS_2_ samples, respectively, which were collected by scanning from 0.1 to 10^6^ Hz with an overpotential of 0.3 V. H_2_-annealed MoS_2_ sample had a smaller semicircle diameter compared to pristine sample, indicating more efficient charge transfer after H_2_ annealing. This also contributes to the enhanced HER activity of H_2_-annealed MoS_2_ samples. The efficient charge transfer could be attributed to the elementary Mo which has better conductivity than MoS_2_, and reduced size of H_2_-annealed MoS_2_ nanosheets. This could explain the higher percentage of S-vacancies than previous calculation, at which the optimum HER activity was achieved.^44,47^ After re-sulfurization, the semicircle diameter of MoS_2_ samples became larger, meaning slower charge transfer than H_2_-annealed MoS_2_ sample. This is also considered to be responsible for the worse HER activity than H_2_-annealed MoS_2_ samples (shown in Fig. 5c). It is also found in Fig. 6a that the semicircle diameter of re-sulfurized MoS_2_ sample was not completely recovered to that of pristine sample, indicating faster charge transfer than pristine sample, which resulted in the partial recovery of HER activity (shown in Fig. 5c). The faster charge transfer could be attributed to the reduced size of re-sulfurized MoS_2_ nanosheets and hence the smaller resistance.

Long-term durability, which demonstrates the thermodynamic stability, is also an important factor to evaluate the overall performance of an electrocatalyst. We investigated the cycling stability of H_2_-annealed vertical MoS_2_ nanosheets sample for 20 min at 780 °C, which had the best electrocatalytic performance. The LSV scanning was performed before and after repeating cycling voltammetry treatment for 10 000 cycles in an acidic environment with a fast scan rate of 100 mV s^−1^ in order to simulate the practical working conditions of water-splitting devices. Fig. 6b shows the cathodic polarization curves of the sample before and after the cycling treatment. It can be seen that there is a negligible change in the cathodic current. Meanwhile, the time-dependent electrochemical measurement for the sample (inset of Fig. 6b) suggests that such electrocatalyst maintained its current density for at least 8 h. Both results indicated the excellent durability of the H_2_-annealed vertical MoS_2_ nanosheets as an electrocatalyst.

## Conclusions

We demonstrated the enhanced HER activity of MoS_2_ nanosheets with vertical configuration and H_2_ annealing. Vertical configuration offered more edge sites compared to horizontal MoS_2_ nanosheets, and hence improved the HER activity. In addition, H_2_ annealing produced a synergistic enhancement in two aspects. First, H_2_ annealing roughened the edges and produced S-vacancies in the basal plane of vertical MoS_2_ nanosheets, which increased the number of active sites. Second, H_2_ annealing leads to the enhancement of the conductivity due to the production of elementary Mo and the decreased sheet size. Moreover, prominent electrochemical durability is also achieved. The H_2_-annealed vertical MoS_2_ nanosheets reported here represents a novel approach for producing highly active nanostructured MoS_2_ as HER catalysts, and can be extended to other applications, such as supercapacitor and lithium-ion battery.

## Conflicts of interest

The authors declare no competing financial interest.

## Supplementary Material

RA-008-C8RA01147H-s001
