# Supplementary material for: Enhanced hydrogen evolution reaction activity of hydrogen-annealed vertical MoS2 nanosheets
Source: RSC Adv. 2018 Apr 17;8(26):14369–76. doi: 10.1039/c8ra01147h (PMC9079879; doi:10.1039/c8ra01147h)
Supplement: RA-008-C8RA01147H-s001 [file RA-008-C8RA01147H-s001.pdf]

Electronic Supplementary Material (ESI) for RSC Advances.
This journal is © The Royal Society of Chemistry 2018

Enhanced Hydrogen Evolution Reaction Activity of Hydrogen-Annealed Vertical MoS_2 Nanosheets

Mengci He,[a] Fanpeng Kong,[b] Geping Yin,[b] Zhe Lv,[c] Xiudong Sun,[a,d] Hongyan Shi,[a,d]* Bo Gao[a,d]*

[a] Institute of Modern Optics, Key Lab of Micro-optics and Photonic Technology of Heilongjiang Province, Key Laboratory of Micro-Nano Optoelectronic Information System, Ministry of Industry and Information Technology, Department of Physics, Harbin Institute of Technology, Harbin 150001, China.

[b] School of Chemistry and Chemical Engineering, Harbin Institute of Technology, Harbin 150001, China.

[c] Department of Physics, Harbin Institute of Technology, Harbin 150080, China

[d] Collaborative Innovation Center of Extreme Optics, Shanxi University, Taiyuan 03006, China.

Corresponding Author:

Prof. Bo Gao

Email: gaobo@hit.edu.cn

Phone: 86-18204505038

Prof. Hongyan Shi

Email: shi.hong.yan@hit.edu.cn

Phone: 86-18646083576

Supplementary Figures

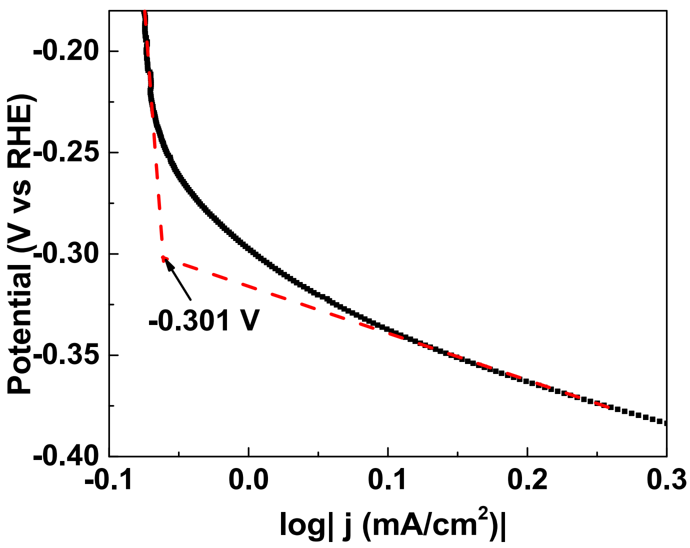

Figure S1. Calculation of onset overpotential using the Tafel plot of the MoS_2 sample in the region of low current densities.

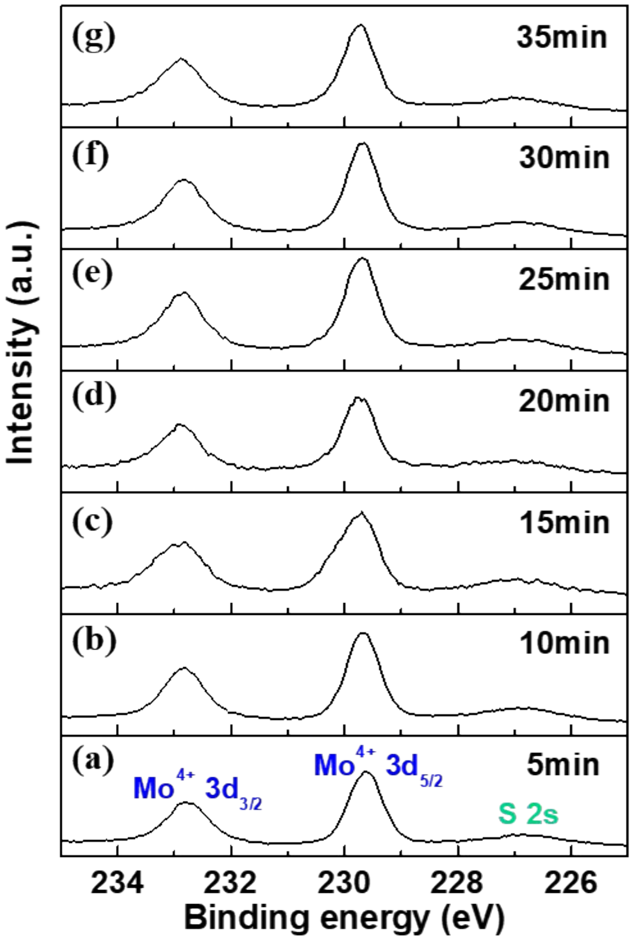

Figure S2. XPS data of Mo 3d region for the re-sulfurized MoS_2 nanosheets for the annealing time of (a) 5, (b) 10, (c) 15, (d) 20, (e) 25, (f) 30 and (g) 35 min, respectively.

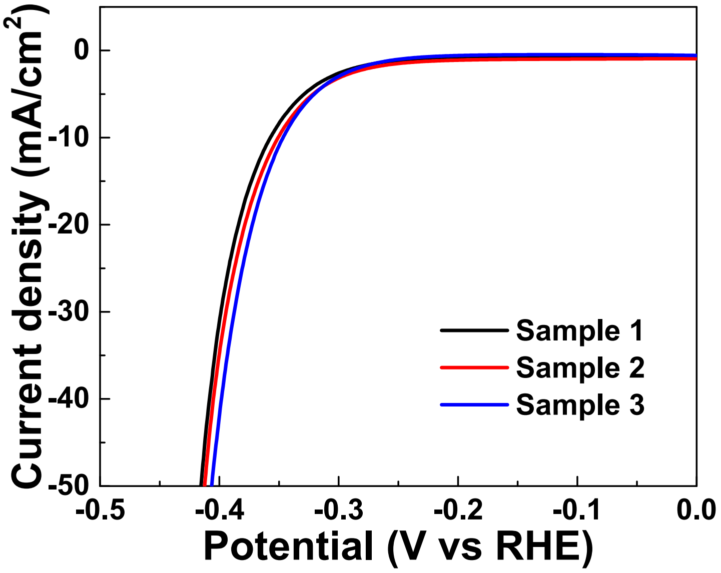

Figure S3. Cathodic polarization curves of three H_2-annealed MoS_2 samples. Annealing time: 20 min.
